# Combined Prokaryotic Transcriptomics and Proteomics Analysis of Clinical *Trueperella pyogenes* Isolates with Distinctive Cytotoxicity

**DOI:** 10.3390/ijms26041490

**Published:** 2025-02-11

**Authors:** Ning Liu, Qian Li, Qiang Shan

**Affiliations:** 1 College of Veterinary Medicine, Northeast Agricultural University, Harbin 150030, China; nliu@neau.edu.cn (N.L.); l33980715@163.com (Q.L.); 2 Key Laboratory of the Provincial Education Department of Heilongjiang for Common Animal Disease Prevention and Treatment, College of Veterinary Medicine, Northeast Agricultural University, Harbin 150030, China

**Keywords:** *Trueperella pyogenes*, virulence, transcriptome, proteomic, genomic analysis

## Abstract

*Trueperella pyogenes* is a widely distributed opportunistic pathogenic bacterium that can infect livestock, wildlife, community animals, and humans, resulting in suppurative infection of tissue and organ mucosa, including pneumonia, liver abscessation, mastitis, metritis, endocarditis, and osteoarthritis. TP1804 and TP1808 were isolated from the uterine lavage fluid of cows with endometritis. This study analyzed the prokaryotic transcriptomics and proteomics of two strains of *T. pyogenes* with similar growth curves but different cytotoxicity. Studying the metabolic mechanisms of these differentially expressed genes and proteins can greatly promote the discovery of new biomarkers and improve the accuracy of biomarker identification, which is of great value for molecular mechanisms, biomarkers, early diagnosis of diseases, molecular typing, and prognosis. Our results indicate that the control of the virulence by tRNAs to bacteria during ribosome biosynthesis is crucial.

## 1. Introduction

*Trueperella pyogenes* is a widely distributed opportunistic pathogenic bacterium that can infect livestock, wildlife, community animals, and humans, resulting in suppurative infection of tissue and organ mucosa, including pneumonia, liver abscessation, mastitis, metritis, endocarditis, and osteoarthritis [1,2,3]. It was reclassified as *T. pyogenes* according to the 16S rRNA sequence based on phylogenetic and chemotaxonomic observations [1,2]. *T. pyogenes* have been isolated from edible animals in many countries around the world [3]. The infection of *T. pyogenes* can cause serious economic losses and even lead to the elimination of infected animals, mainly in breeding cattle and pigs [3,4].

*T. pyogenes* secretes several important virulence factors, such as pyolysin (PLO), fimbriae (Fim), neuraminidase (NanH and NanP), and collagen-binding protein (CbpA) [5,6]. The most important and primary virulence factor is PLO encoded by the *plo* gene [7,8], which is associated with cell lysis and hemolysis [2,9,10]. Other virulence factors are related to adherence, colonization, and pathogenesis [8,11]. Curiously, the previous report exhibited distinct differences in the expression of eight known virulence genes in *T. pyogenes* isolated from cows of different health states [12]. This demonstrates the significance of the regulatory mechanisms of virulence gene expression in the development of infection [8,13]. Liu et al. [3] also reported significant differences in the cell lysis ability of *T. pyogenes* isolated from cow uterine lavage fluid. All this evidence demonstrates that the infection and pathogenicity of *T. pyogenes* may be attributed to other unknown bacterial factors.

Measuring the expression of RNA and proteins in pathogenic bacteria is fundamental for understanding bacterial pathogenicity and pathogenic mechanisms. This study may be the first to explore the pathogenicity and pathogenic mechanism of *T. pyogenes* through the combined analysis of protein levels with transcript levels. These investigations could complement transcriptomic studies and provide insights into post-transcriptional regulatory mechanisms [14], as well as into bacterial pathogenicity and pathogenic mechanisms.

This study analyzed the prokaryotic transcriptomics and proteomics of two strains of *T. pyogenes* with similar growth curves but different cytotoxicity. Our research findings emphasize the importance of comparing transcriptomic and proteomic analyses for bacterial pathogenicity and drug resistance. Our research displayed the differences in bacterial pathogenicity and drug resistance, providing evidence for a better understanding of bacteria virulence genes.

## 2. Results

### 2.1. Bacterial Growth Characteristics and Toxicity

**Figure 1:**
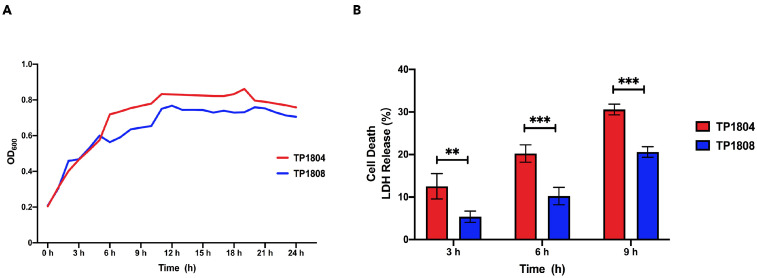
Growth curves (**A**) and cytotoxicity (**B**). (**A**) Bacterial growth curves of TP1804 and TP1808 for 24 h. (**B**) Cytotoxicity of TP1804 and TP 1808 for 3 h, 6 h, and 9 h. The mean ± SEM of data is shown from three independent repeats (** *p* < 0.01, *** *p* < 0.001).

Our previous study reported that 23 strains of *T. pyogenes* were isolated from cow uterine lavage fluid [3]. Among them, TP1804 has the strongest cytotoxicity, while TP1808 has the weakest cytotoxicity. In this study, two strains of *T. pyogenes* with the greatest difference in cytotoxicity were selected to compare their differential genes and proteins. The growth curves of TP1804 and TP1808 were measured from 1 h to 24 h (Figure 1A). The growth curve can reflect the growth condition of bacteria and the time for bacteria to reach the logarithmic phase, which is of great significance. TP1804 and TP1804 both passed their mid-log phase at 11 h with similar growth curves.

The lactate dehydrogenase (LDH) method was performed to examine the cell cytotoxicity after 3 h, 6 h, and 9 h of challenge of TP1804 and TP1808 (Figure 1B). Comparing 3 h, 6 h, and 9 h, the cell death rates infected by TP1804 were particularly higher than those of TP1808. There was no significant difference between TP1804 and TP1808 at 3 h. Starting from 6 h, the cytotoxicity of TP1804 obviously increased and showed significant differences compared to TP1808 (*p* < 0.01).

### 2.2. Sample Correlation and Gene Expression Distribution

**Figure 2:**
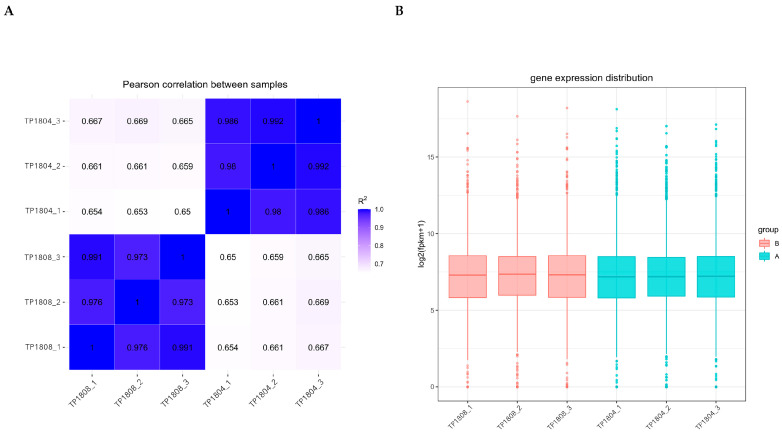
Gene expression correlation (**A**) and gene expression box plot (**B**). (**A**) The horizontal and vertical axes represent the squared correlation coefficients of each sample. (**B**) The horizontal axis represents the sample name, and the vertical axis represents log2 (FPKM + 1).

The correlation between gene expression levels among samples is an important indicator for testing the reliability of experiments and the rationality of sample selection (Figure 2A). The correlation coefficients of between-group and within-group samples were calculated based on the fragments per kilobase million (FPKM) values of all genes in each sample, and a heatmap was drawn to visually display the differences between group samples and the duplication of within-group samples. The higher the correlation coefficient between samples, the closer their expression patterns are. It can be seen that the genes of the samples within the group are very close, while there are differences in the genes of the samples between groups.

Due to the influence of sequencing depth and gene length, RNA-seq gene expression values are generally not represented by read count but by FPKM, which corrects sequencing depth and gene length in sequence. After calculating the expression values (FPKM) of all genes in each sample, the distribution of gene expression levels in different samples is displayed through a box plot (Figure 2B). The box plots for each region show five statistical measures (maximum upper quartile, median, lower quartile, and minimum). The genetic differences within the group are not significant, while the genetic differences between groups are significant.

### 2.3. Differential Gene Analysis

**Figure 3:**
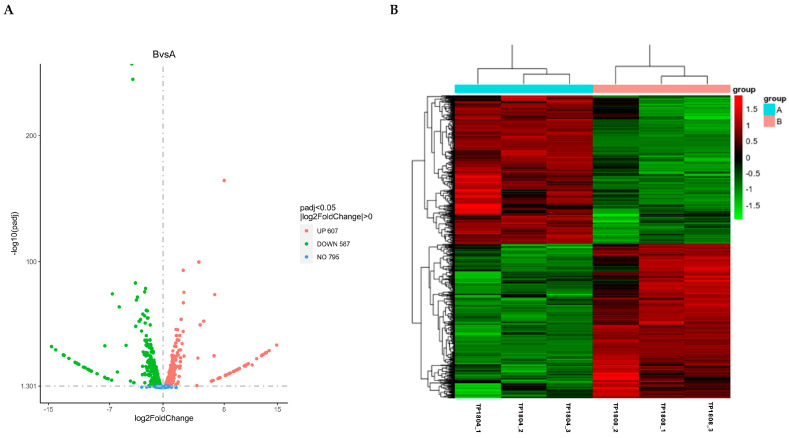
Volcano map of differential gene analysis (**A**) and differential gene clustering heatmap (**B**). (**A**) The horizontal axis presents the log2 (fold change) and the vertical axis represents −log10 (paj) or −log10 (*p*-value); upregulated genes are represented by red dots, while downregulated genes are represented by green dots; (**B**) the horizontal axis represents the sample name and the vertical axis represents the normalized value of the differential gene FPKM.

The volcano map visually displays the distribution of differentially expressed genes for each comparison combination, as shown in Figure 3A. The horizontal axis represents the expression fold change in genes in the treatment and control groups denoted by log2 (Fold Change) and the vertical axis represents the significance level of gene expression differences between the treatment and the control groups denoted by −log10 (paj) or −log10 (*p*-value). There are 607 upregulated genes and 587 downregulated genes. The top ten genes with the most significant upregulation and downregulation are listed in Table 1 and Table 2, respectively.

Cluster analysis of differential genes is set for two or more experiments (Figure 3B). It groups genes with similar expression patterns together, which may have common functions or participate in common metabolic and signaling pathways. Mainstream hierarchical clustering was used to cluster gene expression values and normalize the rows of expression data. Genes or samples with similar expression patterns in the heatmap will be clustered together. The colors in each square do not reflect the gene expression values, but rather the values obtained by normalizing the rows of expression data. Therefore, the colors in the heatmap can only be compared horizontally (the expression of the same gene in different samples), while they cannot be compared vertically (the expression of different genes in the same sample). When comparing horizontally, red indicates high gene expression and green indicates low gene expression.

### 2.4. Enrichment Analysis

**Figure 4:**
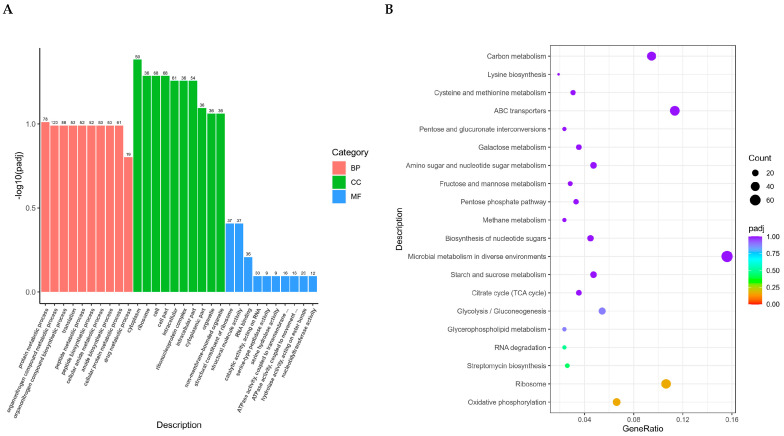
GO function enrichment analysis (**A**) and KEGG pathway enrichment analysis (**B**). (**A**) The horizontal axis represents GO Term and the vertical axis represents the significance level of GO Term enrichment, denoted by −log10 (padj). Different colors indicate different functional classifications. The 30 most significant terms from the GO enrichment analysis results were selected, and a bar chart for display was drawn. If there were less than 30 terms, we drew a bar chart for all terms, classified by biological processes, cellular components, and molecular functions, as well as differentially expressed genes. (**B**) The horizontal axis represents the ratio of the number of differentially expressed genes annotated onto the KEGG pathway to the total number of differentially expressed genes, while the vertical axis represents the KEGG pathway.

ClusterProfiler software virsion 4.6.1 as used to perform GO functional enrichment analysis and KEGG pathway enrichment analysis on differential gene sets (Figure 4). It shows the enrichment analysis results of all significantly different genes for all combinations. The differential gene set is obtained from significant difference analysis and annotated to GO or KEGG databases, while the background gene set is of all genes subjected to significant difference analysis and annotated to GO or KEGG databases. The enrichment analysis results enrich all sets of differentially expressed genes, upregulated differentially expressed genes, and downregulated differentially expressed genes for each differential comparison combination.

The 30 most significant terms from the GO enrichment analysis results were selected (Figure 4A). The candidate genes cover the ten most significant differences in biological process (BP), cellular component (CC), and molecular function (MF), respectively. The 20 most significant KEGG pathways from the KEGG enrichment results were selected and a scatter plot was drawn (Figure 4B). The results showed that six pathways reached the recommended significant level, which were oxidative phosphorylation, ribosome, streptomycin biosynthesis, RNA degradation, glycerophospholipid metabolism, and glycolysis/gluconeogenesis. Through the analysis of gene enrichment results of GO and KEGG, it can be seen that these genes are related to the bacterial biological process.

### 2.5. Principal Component Analysis (PCA)

**Figure 5:**
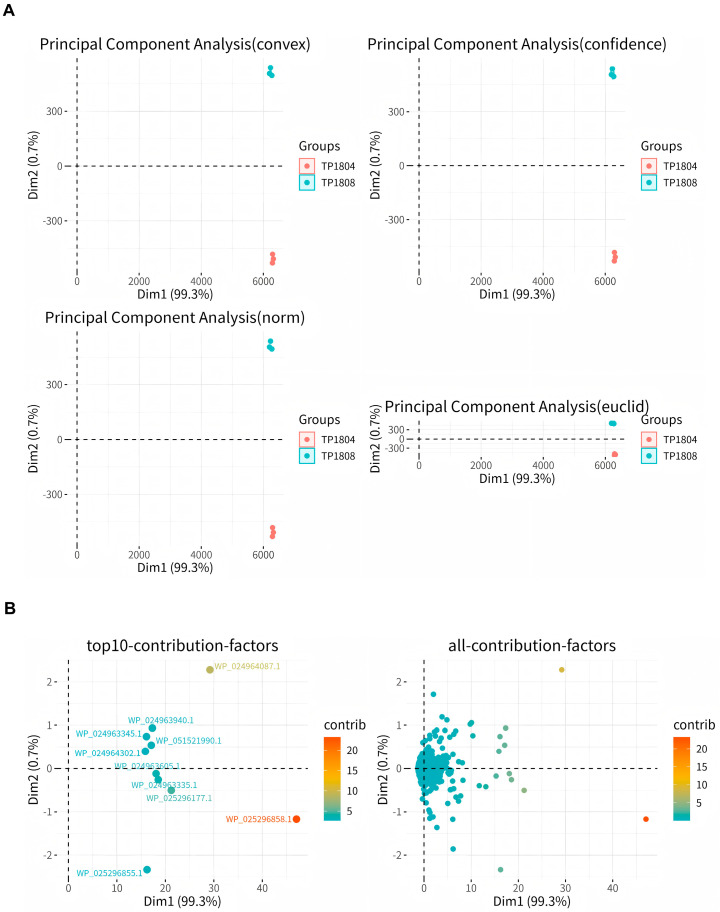
Sample grouping PCA analysis (**A**) and the principal component diagram of the top ten contributions (**B**).

PCA is the process of dimensionality reduction for multiple variables into a few independent variables (Figure 5). In proteomic analysis, PCA reduces the large amount of protein expression information contained in the sample into a few independent principal components for comparison between groups, facilitating the identification of outlier samples and discrimination of sample clusters with high similarity. In Figure 5A, the results show that samples within groups gather together, while samples between groups separate. There are significant differences in the genes of two different clinical *T. pyogenes* isolates. In Figure 5B, we show the top 10 proteins with significant differences between groups for analysis, which are 50S ribosomal proteinL7/L12, phosphopyruvate hydratase, type I plyceraldehyde-3-phosphate dehydrogenase, elongation factor G, formate C-acetyltransferase, phosphoenolpyruvate carboxykinase (GTP), class II fructose-bisphosphate aldolase, type I polyketide synthase, elongation factor Tu, and chaperonin GroEL.

### 2.6. Differential Expression Protein Analysis

**Figure 6:**
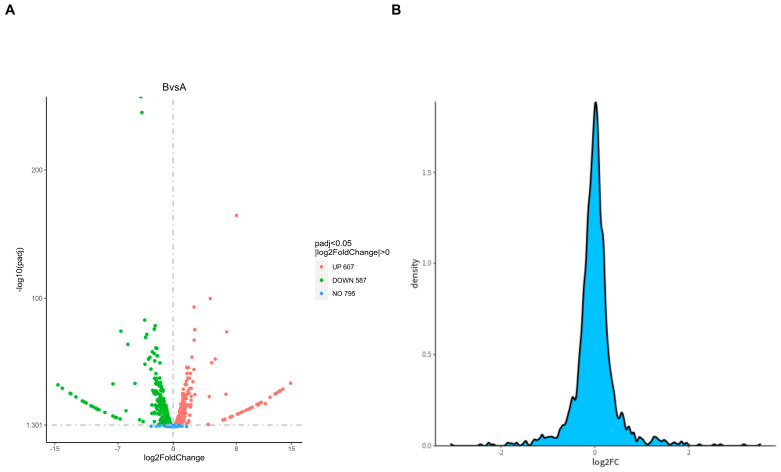
Differential protein volcano diagram (**A**) and differential multiple density plot (**B**). (**A**) The horizontal axis represents the expression fold changes in proteins in different experimental groups/samples, and the vertical axis represents the statistical significance of changes in protein expression levels. (**B**) The X-axis represents log2 (FC) and the Y-axis represents the fold density of proteins between groups.

For sample analysis with biological replicates, DESeq2 was conducted based on the negative binomial distribution (Figure 6A). By comparing each sample group pairwise, we were able to identify the proteins with differential expression in different groups. The scattered dots in the figure represent proteins. Gray dots represent proteins with no significant differences. Red dots represent significantly upregulated differential proteins. Green dots represent significantly downregulated differential proteins. The top ten proteins with the most significant upregulation and downregulation are listed in Table 3 and Table 4, respectively. Figure 6B shows the ratio of the number of proteins to the total number at this difference multiple. In theory, the vast majority of proteins have no significant differences, so the peak position of FC should be around 0 and show a normal distribution.

### 2.7. Differential Protein Functional Enrichment Analysis

**Figure 7:**
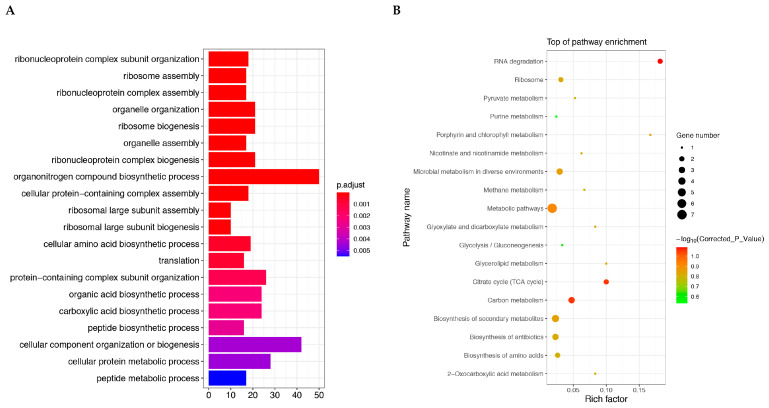
Candidate protein GO function enrichment analysis (**A**) and KEGG pathway enrichment analysis (**B**). (**A**) The vertical axis represents the GO term at the next level of the three major categories and the horizontal axis represents the number of candidate proteins annotated under that term (including its subterms). (**B**) The top 20 enriched pathway entries were selected for display in (**B**). If there are less than 20 enriched pathway entries, they will all be displayed. *p*-value is the corrected *p*-value.

Based on the identification of all differentially expressed proteins, we conducted enrichment analysis using GO and KEGG, with the aim of detecting whether differentially expressed proteins have significant enrichment trends in certain functional types. The differential protein GO enrichment results are shown in Figure 7A containing the top 20 sub-functions with highly significant corrected *p*-values. The highest gene ratio is the term of the organonitrogen compound biosynthetic process.

In organisms, different genes coordinate with each other to perform their biological functions, and significant enrichment through pathways can determine the most important biochemical metabolic pathways and signal transduction pathways involved in candidate proteins. The KEGG enrichment scatter plot of candidate proteins is a graphical display of KEGG enrichment analysis results (Figure 7B). The top 10 most significant terms are RNA degradation, citrate cycle, carbon metabolism, metabolic pathways, biosynthesis of secondary metabolites, microbial metabolism in diverse environments, porphyrin and chlorophyll metabolism, glycerolipid metabolism, biosynthesis of antibiotics, and ribosome.

## 3. Discussion

Analyzing the genetic characteristics of different clinical strains of *T. pyogenes* can better understand the pathogenic mechanism of *T. pyogenes*. This study used *T. pyogenes* with significant differences in virulence isolated from the same dairy farm as research materials, combined with genome and proteome analysis, to explore their genetic characteristics and pathogenic mechanisms. This study mainly includes the determination of growth curves and the cytotoxicity of two clinical strains of *T. pyogenes*, genomic analysis of differential genes between two strains of *T. pyogenes*, and differential proteins coded by them based on proteomics analysis.

Our previous studies have shown that both TP1804 and TP1808 express genes of *plo*, *nanP*, *cbpA*, *fimA*, *fimC*, and *fimE* [3]. These virulence factors contribute to promoting adhesion, including fimbriae, extracellular matrix-binding proteins, and neuraminidases [1]. Fimbriae, including FimA, FimB, FimC, FimE, and FimG, are involved in the adhesion and colonization of host tissue [15]. Collagen-binding protein A (CbpA) is also expressed by *T. pyogenes* to associate with adhesion and improve colonization by binding to extracellular matrix compounds on the surface of mammalian cells [16]. The expression levels of virulence genes are of importance to the pathogenicity of *T. pyogenes* [17]. However, the creation and advancement of the *T. pyogenes* infection may originate not only from these known factors but also from other unknown virulence factors [3,8]. It is frustrating that these unknown virulence factors have been rarely studied in depth.

Moreover, the prokaryotic transcriptome results have shown that three tRNAs showed significant differences between the two strains of *T. pyogenes*. The differences in tRNA mainly manifest in the initial stage of protein synthesis, involving the interaction between tRNA and ribosome translation mechanism components [18]. In eukaryotes, the initiator methyonyl-tRNA (Met-tRNAi) will not be formylated [19]. However, whether this phenomenon also applies to prokaryotes remains to be studied. This may be the main factor affecting the virulence of bacteria. As in canonical translation, the nascent chain is transferred to the Ala residue of SsrA/transfer-messenger RNA (tmRNA) in the peptidyl transferase center (PTC) and translation resumes on the open reading frame (ORF) of the SsrA mRNA-like domain [20]. Amazingly, the *plo* gene, which encodes the PLO toxin, is located in the ORF [6]. This indicates that the virulence of different clinical strains of *T. pyogenes* is closely related to the ribosomes and their biosynthesis.

Many RNA modifications were first discovered on tRNA. It is known that tRNA has the highest frequency of modifying nucleosides per length. There is an average of eight modifications per tRNA molecule in bacteria [21]. In bacteria, tRNA modification is essential for responding to stress and regulating the expression of virulence factors. It is a participant in bacterial and viral infections, such as regulating bacterial growth, adapting to stress conditions and host antiviral responses, and affecting viral replication and pathogenicity [22,23]. About 1.5% of the proteins in the *E. coli* proteome are required for the synthesis of different tRNA nucleotide modifications, which also shows the importance of tRNA modification in cell function [24,25]. tRNA modification plays an important role in the virulence of pathogenic bacteria. In *Pseudomonas aeruginosa*, tRNA-modifying enzymes are required to induce cytotoxicity in human lung epithelial cells [26]. The expression of Ttc A is upregulated under oxidative stress conditions, and the absence of this enzyme in the host model will affect virulence [27]. The deletion of Trm B affects the translation of tRNAPhe (GAA) and tRNAAsp (GUC) codon-enriched transcripts, including catalase, which decomposes hydrogen peroxide, KatA, and KatB, thereby affecting virulence [28].

In the current study, we conducted prokaryotic transcriptomics and proteomics to explore the genetic characteristics between two different strains of *T. pyogenes*. We found that three tRNAs showed significant differences between the two strains of *T. pyogenes*. However, the limitation of this study is that only two strains were investigated. More strains should be involved in future research. Determining how the control of virulence by tRNA to bacteria functions during ribosome biosynthesis also requires further study. Studying the metabolic mechanisms of these differentially expressed genes and proteins can largely stimulate the development of new biomarkers and enhance the accuracy of biomarker identification, which is of extreme importance for molecular mechanisms, biomarkers, early diagnosis of diseases, molecular typing, and prognosis.

## 4. Materials and Methods

### 4.1. Strain and Culture Medium

The strains of *T. pyogenes* TP1804 and TP1808 used in this study were isolated from dairy cow uterine lavage fluid and stored in 25% glycerol at −80 °C. Sheep blood culture medium and Brain–Heart Infusion (BHI) Broth were used for bacterial isolation and activation. The growth conditions of the two strains of bacteria are completely identical.

### 4.2. DNA Extraction and Sequencing

The DNA of *T. pyogenes* was extracted and the 16S gene was sequenced, as previously described [29]. Briefly, the DNA was extracted with a Solarbio bacterial DNA kit (D3350, Solarbio, Beijing, China), and PCR amplification was performed by 16S gene primers (F: AACTGGAGGAAGGTGGGGAT, R: AGGAGGTGATCCAACCGCA). The PCR products were sent to Sangon Biotech (Shanghai, China) Co., Ltd. for sequencing, and the results were analyzed using the BLAST program (National Center for Biotechnology Information, Bethesda, MD, USA).

### 4.3. Bacterial Growth Curves

To examine different growth rates of *T. pyogenes*, bacterial growth curves were measured for TP1804 and TP1808 in BHI Broth with a Turbidity meter (BioMérieux, Durham, NC, USA) as previously described [1]. The 96-well plate was put in the microplate reader (Tecan Group, Männedorf, Switzerland) at 37 °C, and OD_600_ was measured every hour for a continuous 24 h of measurement.

### 4.4. Cell Culture

The primary bovine endometrial epithelial cells (BEECs) were purchased from BeNa Culture Collection, Beijing, China. The BEECs were cultured with Dulbecco’s Modified Eagle medium/Ham’s F-12 medium (1:1) (Invitrogen, Carlsbad, CA, USA) and supplied with 10% heat-inactivated fetal horse serum (Invitrogen, Carlsbad, CA, USA) and 1% penicillin and streptomycin (Invitrogen, Carlsbad, CA, USA). After flowing evenly into cell flasks (Corning, Corning, NY, USA), the BEECs were cultured at 37 °C in an incubator with 5% CO_2_ atmosphere.

### 4.5. Lactate Dehydrogenase (LDH) Assay

The cytotoxicity was evaluated with LDH Cytotoxicity Assay (Beyotime, Beijing, China) following the instructions of the manufacturer. BEECs (2 × 10^5^ cells/well) were harvested into six-well cell culture plates (Corning, Corning, NY, USA) after reaching 80% confluency as previously described [30]. The release of LDH into the supernatant from the control cells (Medium alone) and cells challenged by TP1804 and TP1808 (1 × 10^6^ CFU/well) for 9 h was measured. A maximum-lysis control was used for uninfected cells. Cells were washed three times with PBS and incubated with 100 μL Trypsin-EDTA (0.25%, Gibico, Waltham, MA, USA) for 4 min after the supernatant was collected for LDH measurement and bacteria counting. F-12 medium (1:1, 900 μL) was added into each well and pipetted completely for cell counting. The results of cytotoxicity or mortality (%) were calculated using the following formula: % cytotoxicity = (infected LDH − control LDH)/(max lysis LDH − correction volume LDH − control LDH + background LDH) × 100%.

### 4.6. RNA Extraction and Transcriptomic Analysis

Total RNA was extracted with an RNAprep Pure Cell/Bacterial Kit (TIangen Biotech Co., Ltd, Beijing, China) following the manufacturer’s instruction [31]. The concentration of bacteria for RNA extraction exceeded 1 × 10^6^ CFU. The purity and integrity of total RNA were analyzed by agarose gel electrophoresis, Nanodrop (OD 260/280 ratio), Qubit 2.0, and Agilent 2100. A TruSeqTM Stranded Total RNA Library Prep Kit was used to generate sequencing libraries. Sequencing was carried out by Wekemo Tech Co., Ltd. (Shenzhen, China) on the Illumina (Miseq/Hiseq) platform. The quality control on the raw sequencing data of each sample used FastP software v0.23.4, and clean data were obtained for downstream analysis.

The Bowtie2 software v2.5.2 was used to perform genomic localization analysis on the filtered sequences [32]. If the reference genome selection is appropriate and there is no contamination in the relevant experiments, the proportion of sequencing reads successfully aligned with the genome generated by the experiment will be higher than 70% (Total Mapped Reads or Fragments), with the percentage of sequencing sequences with multiple loci (Multiple Mapped Reads or Fragments) usually not exceeding 10% of the total. ClusterProfiler software virsion 4.6.1 was used to perform Gene Ontology (GO) functional enrichment analysis and the Kyoto Encyclopedia of Genes and Genomes (KEGG) pathway enrichment analysis on differential gene sets. The differential gene set is obtained from significant difference analysis and annotated to GO or KEGG databases, while the background gene set is of all genes subjected to significant difference analysis and annotated to GO or KEGG databases. The enrichment analysis results enrich all sets of differentially expressed genes, upregulated differentially expressed genes, and downregulated differentially expressed genes for each differential comparison combination. After the quantification of gene expression is completed, statistical analysis of its expression data is required to screen for genes with significant differences in expression levels in different states of the sample.

### 4.7. Proteomic Analysis

Protein was extracted as previously described [31]. Briefly, each sample was washed three times with sterilized PBS, and 1 mL of radio-immunoprecipitation assay (RIPA, Solarbio, Beijing, China) buffer containing 1% phenylmethylsulfonyl fluoride (PMSF, Solarbio, Beijing, China) was added to each sample and ground for 5 min at 4 °C. The mixture was centrifuged at 12,000× *g* for 15 min at 4 °C after incubating with rotation at 4 °C for 10 min. The nano LC-MS/MS analysis of peptides was performed by Wekemo Tech Co., Ltd. (Shenzhen, China). Based on the identification of all differentially expressed proteins, enrichment analyses were conducted through GO and KEGG to detect whether differentially expressed proteins have significant enrichment trends in certain functional types. The experiment was repeated three times.

### 4.8. Statistical Analysis

All experiments were repeated three times, and data were exhibited as mean values with standard deviations. The screening criteria for significantly differentially expressed genes were FDR < 0.05, with the additional criteria of |log2 (fold change)| > 1. The differentially expressed proteins were screened by the criteria of fold change > 1.2 (over 1.2-fold upregulation) or <0.83 (downregulation), and *p* < 0.05 was considered statistically significant [33].

## 5. Conclusions

The main hemolysin of *T. pyogenes* is PLO, which is also recognized as a cytotoxic agent. Although all *T. pyogenes* strains express PLO, the cytotoxicity exhibited by different strains varies. Following a deep comparison between two strains of *T. pyogenes* with significant differences in virulence through prokaryotic transcriptomics and proteomics, three tRNAs showed significant differences between the two strains of *T. pyogenes*. Our results indicate that *T. pyogenes* strains with different virulence have significant differences in tRNAs during ribosome biosynthesis. However, more detailed studies on the mechanisms of virulence are still needed.

## Figures and Tables

**Table 1:** Upregulated gene statistics results (Top 10).

ID/Name	Description	Log2 FC	*p*-Value
CQ11_RS06575	hypothetical protein	8.01881517709806	6.24680407290023 × 10^−168^
CQ11_RS07205	choice-of-anchor M domain-containing protein	4.69405827857662	3.98052216466355 × 10^−103^
CQ11_RS00725	hypothetical protein	2.6437161209102	1.7332343504935 × 10^−96^
CQ11_RS07305	HD domain-containing protein	2.73722865310865	1.07719646567085 × 10^−78^
CQ11_RS11205	DUF1349 domain-containing protein	6.77907877522527	7.36392725346365 × 10^−77^
CQ11_RS00105	rhodanese-like domain-containing protein	2.65861336514393	3.45483260566808 × 10^−70^
CQ11_RS08465	dimethyl sulfoxide reductase anchor subunit	2.37509451739112	6.22678372311554 × 10^−57^
CQ11_RS05835	hypothetical protein	5.32834123917815	2.18027298280556 × 10^−55^
CQ11_RS10335	tRNA-Met	4.86792626174594	2.23813376131865 × 10^−52^
CQ11_RS02580/*pheT*	Phenylalanine tRNA ligase subunit beta/Ferredoxin-fold anticodon binding domain	1.70567200296519	1.02370047692426 × 10^−48^

**Table 2:** Downregulated gene statistics results (Top 10).

ID/Name	Description	Log2 FC	*p*-Value
CQ11_RS04815	peptidoglycan bridge formation glycyltransferase FemA/FemB family protein	−4.09598434309614	0
CQ11_RS04810	UDP-N-acetylmuramate dehydrogenase/UDP-N-acetylenolpyruvoylglucosamine reductase	−3.96207095264488	2.17463477730769 × 10 ^−248^
CQ11_RS04820/*rpmG*	50S ribosomal protein L33	−3.61149921577885	2.49897333558599 × 10 ^−86^
CQ11_RS06175	universal stress protein	−2.28635433173304	7.53326393741449 × 10 ^−82^
CQ11_RS04790	phosphotransferase	−2.39848722472287	3.50225282301118 × 10 ^−79^
CQ11_RS05950	hypothetical protein	−6.63226646505396	1.89968005619395 × 10 ^−77^
CQ11_RS04825	tRNA-Met	−3.35664880655572	9.57703127155594 × 10 ^−75^
CQ11_RS07665	type IV toxin-antitoxin system AbiEi family antitoxin domain-containing protein	−3.49604862585357	2.04467148122348 × 10 ^−72^
CQ11_RS03615	tRNA-Glu	−5.74227957327654	6.64897669722015 × 10 ^−67^
CQ11_RS01540	RNA-binding S4 domain-containing protein	−2.20257447774072	3.71201280550628 × 10 ^−64^

**Table 3:** Upregulated protein statistics results (Top 10).

ID/Name	Description	Log2 FC	*p*-Value
WP_024963008.1	sulfatase-like hydrolase/transferase	0.976030237642828	1.02255789360905 × 10^−53^
WP_024963088.1	hypothetical protein	0.48038724806113	9.16585334898564 × 10^−21^
WP_024963127.1/pknB	Stk1 family PASTA domain-containing Ser/Thr kinase	0.0682783903146863	0.016432574900883
WP_024963128.1	penicillin-binding protein	0.069473085083476	0.0497705393613076
WP_024963167.1	threonylcarbamoyl-AMP synthase	0.183202749033643	0.0000800968930751222
WP_024963168.1	hypothetical protein	0.19200766313386	0.000144392977367676
WP_024963245.1	dCTP deaminase	0.128853301697393	0.0114288489838083
WP_024963273.1/sufB	Fe-S cluster assembly protein SufB	0.0871993586367944	0.000183042840201825
WP_024963278.1	SUF system NifU family Fe-S cluster assembly protein	0.797769603520733	4.35880831787613 × 10^−15^
WP_024963289.1	NfeD family protein	0.441682051391175	1.42689523273307 × 10^−8^

**Table 4:** Downregulated protein statistics results (Top 10).

ID/Name	Description	Log2 FC	*p*-Value
WP_024963019.1	DUF3039 domain-containing protein	−0.296962054944321	0.0000746811041672553
WP_024963030.1	peroxiredoxin	−0.319549484183203	7.9950611280789 × 10^−27^
WP_024963103.1	ferredoxin family protein	−0.179468051587051	0.00566840136579069
WP_024963105.1	citrate synthase	−0.415582660837812	5.97700695284514 × 10^−67^
WP_024963116.1	DNA topoisomerase (ATP-hydrolyzing) subunit B	−0.127299178762501	0.0000394313454208064
WP_024963121.1	hypothetical protein	−0.24636030814353	0.0398927209768755
WP_024963122.1	peptidylprolyl isomerase	−0.0830256030105712	0.000222111411178628
WP_024963141.1	ribonuclease HI	−0.13186639640718	0.0419950883999149
WP_024963170.1	DUF3145 domain-containing protein	−0.343841482144734	0.0231910525838472
WP_024963187.1	deoxyguanosinetriphosphate triphosphohydrolase	−0.0796591823712023	0.0354396623845714

## Data Availability

All experimental data are included in this article.

## References

[1] Zhang Z., Liang Y., Yu L., Chen M., Guo Y., Kang Z., Qu C., Tian C., Zhang D., Liu M. (2021). TatD DNases Contribute to Biofilm Formation and Virulence in Trueperella pyogenes. Front. Microbiol..

[2] Qin L., Meng F., He H., Yang Y.B., Wang G., Tang Y.D., Sun M., Zhang W., Cai X., Wang S. (2021). A Virulent Trueperella pyogenes Isolate, Which Causes Severe Bronchoconstriction in Porcine Precision-Cut Lung Slices. Front. Vet. Sci..

[3] Liu N., Shan Q., Wu X., Xu L., Li Y., Wang J., Wang X., Zhu Y. (2024). Phenotypic Characteristics, Antimicrobial Susceptibility and Virulence Genotype Features of Trueperella pyogenes Associated with Endometritis of Dairy Cows. Int. J. Mol. Sci..

[4] Risseti R.M., Zastempowska E., Twaruzek M., Lassa H., Pantoja J.C.F., de Vargas A.P.C., Guerra S.T., Bolanos C.A.D., de Paula C.L., Alves A.C. (2017). Virulence markers associated with Trueperella pyogenes infections in livestock and companion animals. Lett. Appl. Microbiol..

[5] Ashrafi Tamai I., Mohammadzadeh A., Zahraei Salehi T., Mahmoodi P. (2018). Genomic characterisation, detection of genes encoding virulence factors and evaluation of antibiotic resistance of Trueperella pyogenes isolated from cattle with clinical metritis. Antonie Van Leeuwenhoek.

[6] Shan Q., Ma W., Li B., Li Q., Wang X., Li Y., Wang J., Zhu Y., Liu N. (2024). Revealing the Mechanism of NLRP3 Inflammatory Pathway Activation through K(+) Efflux Induced by PLO via Signal Point Mutations. Int. J. Mol. Sci..

[7] Tamai I.A., Mohammadzadeh A., Mahmoodi P., Pakbin B., Salehi T.Z. (2023). Antimicrobial susceptibility, virulence genes and genomic characterization of Trueperella pyogenes isolated from abscesses in dairy cattle. Res. Vet. Sci..

[8] Rzewuska M., Kwiecien E., Chrobak-Chmiel D., Kizerwetter-Swida M., Stefanska I., Gierynska M. (2019). Pathogenicity and Virulence of Trueperella pyogenes: A Review. Int. J. Mol. Sci..

[9] Zhao K., Liu M., Zhang X., Wang H., Yue B. (2013). In vitro and in vivo expression of virulence genes in Trueperella pyogenes based on a mouse model. Vet. Microbiol..

[10] Jost B.H., Billington S.J. (2005). Arcanobacterium pyogenes: Molecular pathogenesis of an animal opportunist. Antonie Van Leeuwenhoek.

[11] Ashrafi Tamai I., Mohammadzadeh A., Zahraei Salehi T., Mahmoodi P., Pakbin B. (2021). Investigation of antimicrobial susceptibility and virulence factor genes in Trueperella pyogenes isolated from clinical mastitis cases of dairy cows. Food Sci. Nutr..

[12] Ibrahim M., Peter S., Wagener K., Drillich M., Ehling-Schulz M., Einspanier R., Gabler C. (2017). Bovine Endometrial Epithelial Cells Scale Their Pro-inflammatory Response In vitro to Pathogenic Trueperella pyogenes Isolated from the Bovine Uterus in a Strain-Specific Manner. Front. Cell Infect. Microbiol..

[13] Rudnick S.T., Jost B.H., Billington S.J. (2008). Transcriptional regulation of pyolysin production in the animal pathogen, Arcanobacterium pyogenes. Vet. Microbiol..

[14] Jiang L., Wang M., Lin S., Jian R., Li X., Chan J., Dong G., Fang H., Robinson A.E., Consortium G.T. (2020). A Quantitative Proteome Map of the Human Body. Cell.

[15] Liu M., Wang B., Liang H., Ma B., Wang J., Zhang W. (2018). Determination of the expression of three fimbrial subunit proteins in cultured Trueperella pyogenes. Acta Vet. Scand..

[16] Pietrocola G., Valtulina V., Rindi S., Jost B.H., Speziale P. (2007). Functional and structural properties of CbpA, a collagen-binding protein from Arcanobacterium pyogenes. Microbiology.

[17] Alkasir R., Wang J., Gao J., Ali T., Zhang L., Szenci O., Bajcsy A.C., Han B. (2016). Properties and antimicrobial susceptibility of Trueperella pyogenes isolated from bovine mastitis in China. Acta Vet. Hung..

[18] Graifer D., Karpova G. (2015). Interaction of tRNA with eukaryotic ribosome. Int. J. Mol. Sci..

[19] Hinnebusch A.G. (2011). Molecular mechanism of scanning and start codon selection in eukaryotes. Microbiol. Mol. Biol. Rev..

[20] Filbeck S., Cerullo F., Pfeffer S., Joazeiro C.A.P. (2022). Ribosome-associated quality-control mechanisms from bacteria to humans. Mol. Cell.

[21] Zhang W., Foo M., Eren A.M., Pan T. (2022). tRNA modification dynamics from individual organisms to metaepitranscriptomics of microbiomes. Mol. Cell.

[22] Mandler M.D., Maligireddy S.S., Guiblet W.M., Fitzsimmons C.M., McDonald K.S., Warrell D.L., Batista P.J. (2024). The modification landscape of Pseudomonas aeruginosa tRNAs. RNA.

[23] Srinivasan R., Ramadoss R., Kandasamy V., Ranganadin P., Green S.R., Kasirajan A., Pillai A.B. (2025). Exploring the regulatory role of small RNAs in modulating host-pathogen interactions: Implications for bacterial and viral infections. Mol. Biol. Rep..

[24] Bjork G.R., Hagervall T.G. (2014). Transfer RNA Modification: Presence, Synthesis, and Function. EcoSal Plus.

[25] de Crécy-Lagard V., Ross R.L., Jaroch M., Marchand V., Eisenhart C., Brégeon D., Motorin Y., Limbach P.A. (2020). Survey and Validation of tRNA Modifications and Their Corresponding Genes in sp Subtilis Strain 168. Biomolecules.

[26] Lin Q., Huang J., Liu Z., Chen Q., Wang X., Yu G., Cheng P., Zhang L.H., Xu Z. (2022). tRNA modification enzyme MiaB connects environmental cues to activation of Pseudomonas aeruginosa type III secretion system. PLoS Pathog..

[27] Romsang A., Duang-Nkern J., Khemsom K., Wongsaroj L., Saninjuk K., Fuangthong M., Vattanaviboon P., Mongkolsuk S. (2018). Pseudomonas aeruginosa ttcA encoding tRNA-thiolating protein requires an iron-sulfur cluster to participate in hydrogen peroxide-mediated stress protection and pathogenicity. Sci. Rep..

[28] Thongdee N., Jaroensuk J., Atichartpongkul S., Chittrakanwong J., Chooyoung K., Srimahaeak T., Chaiyen P., Vattanaviboon P., Mongkolsuk S., Fuangthong M. (2019). TrmB, a tRNA m7G46 methyltransferase, plays a role in hydrogen peroxide resistance and positively modulates the translation of katA and katB mRNAs in Pseudomonas aeruginosa. Nucleic Acids Res..

[29] Liu N., Wang X., Shan Q., Xu L., Li Y., Chu B., Yang L., Wang J., Zhu Y. (2022). Lactobacillus rhamnosus Ameliorates Multi-Drug-Resistant Bacillus cereus-Induced Cell Damage through Inhibition of NLRP3 Inflammasomes and Apoptosis in Bovine Endometritis. Microorganisms.

[30] Shan Q., Liu N., Wang X., Zhu Y., Yin J., Wang J. (2022). Lactobacillus rhamnosus GR-1 attenuates foodborne Bacillus cereus-induced NLRP3 inflammasome activity in bovine mammary epithelial cells by protecting intercellular tight junctions. J. Anim. Sci. Biotechnol..

[31] Zhao Y., Min H., Luo K., Zhang R., Chen Q., Chen Z. (2022). Transcriptomics and proteomics revealed the psychrotolerant and antibiotic-resistant mechanisms of strain Pseudomonas psychrophila RNC-1 capable of assimilatory nitrate reduction and aerobic denitrification. Sci. Total Environ..

[32] Langmead B., Salzberg S.L. (2012). Fast gapped-read alignment with Bowtie 2. Nat. Methods.

[33] Liu S., Liu Y., Zhang J. (2021). Proteomic mechanisms for the regulation of growth, photosynthetic activity and nitrogen fixation in *Nostoc* sp. PCC 7120 exposed to three antibiotic contaminants. Ecotoxicol. Environ. Saf..

